# Cytotoxic effects of docetaxel as a candidate drug of drug-eluting stent on human umbilical vein endothelial cells and the signaling pathway of cell migration inhibition, adhesion delay and shape change

**DOI:** 10.1093/rb/rbx010

**Published:** 2017-05-05

**Authors:** Tingzhang Hu, Chun Yang, Meiling Fu, Jiali Yang, Rolin Du, Xiaolin Ran, Tieying Yin, Guixue Wang

**Affiliations:** Key Laboratory for Biorheological Science and Technology of Ministry of Education, State and Local Joint Engineering Laboratory for Vascular Implants, Bioengineering College of Chongqing University, Chongqing 400030, China

**Keywords:** cytotoxic effects, docetaxel, endothelial cells, signaling pathway

## Abstract

Docetaxel (DTX), a paclitaxel analogue, can efficiently inhibit proliferation of vascular smooth muscle cells and has broadly been used as an antiangiogenesis drug. However, as a candidate drug of drug-eluting stent, the effects of DTX on human umbilical vein endothelial cells (HUVECs) are still not well understood. Herein, we investigated the effects of DTX on proliferation, apoptosis, adhesion, migration and morphology of HUVECs *in vitro*. We found that DTX had the cytostatic and cytotoxic effects at low and high concentrations, respectively. DTX could inhibit the proliferation and migration of HUVECs, induce HUVECs apoptosis, delay HUVECs adhesion and decrease spreading area and aspect ratio of individual cells. The signaling pathway that DTX led to the migration inhibition, adhesion delay and shape change of HUVECs is the VE-cadherin mediated integrin β1/FAK/ROCK signaling pathway. The study will provide a theoretical basis for the clinical application of DTX.

## Introduction

Among various vascular diseases, the cardiovascular disease, especially coronary artery disease (CAD), is by far the leading cause of mortality worldwide [1]. The World Health Organization (WHO) speculated that the global number of deaths from CAD will rise to 2.34 × 10^8^ people by 2030 [2, 3]. Drug-eluting stent (DES) is the most potential method for the treatment of the disease, especially in inhibiting vascular restenosis. The DES has dramatically reduced the incidence of in-stent restenosis [4]. The restenosis rate was reduced to 3–20% after DES implantation [5]. But the subsequent clinical manifestation is astonishing, that is stent thrombosis and endothelial delay problems attendant [6, 7]. The main factor leading to the adverse reactions is drug use of DES. The drugs often do not have cell specificity, which not only inhibits vascular smooth muscle cell (VSMC) proliferation and migration but also inhibits vascular endothelial cell (VEC) proliferation and migration. The inhibition of VECs proliferation often delayed or even suppressed endothelial repair, causing vascular inflammation and neoatherosclerosis [8]. Therefore, the drug selection of DES is important.

Paclitaxel (PTX) and its derivatives are lipophilic drugs, which have broadly been used as anti-proliferative drugs [9]. Currently, PTX is used as the drug for available DES [10]. Docetaxel (DTX), a PTX analogue, is a semisynthetic product generated by esterification of 10-deacetylbaccatin III. 10-Deacetylbaccatin III is isolated and purified from the needles of the European yew tree (*Taxus baccata*) [11]. DTX is a microtubule-stabilizing agent and has been approved for the treatments of breast cancer, prostate cancer and small cell carcinoma of the lung. DTX and PTX are very similar and belong to the same antineoplastic class of compounds. They bind to β tubulin as a tubulin assembly promoter and microtubule stabilizer and cause cell-cycle arrest. DTX is approximately 2-fold more potent than PTX [12, 13]. PTX has two distinct effects on human endothelial cell (EC): one is the cytostatic effect at low concentrations and the other is the cytotoxic effect at high concentrations.

The cytotoxic effect is primarily related to the damage of signaling networks in tumor cells, including microtubule network disturbance, mitochondria permeabilization, G2-M arrest, and increase in the Bax/Bcl-2 ratio, which leads to apoptosis [14, 15]. On the contrary, the cytostatic effect of PTX is associated with the inhibition of EC proliferation, but do not induce apoptosis and the structural changes of the microtubule network. Moreover, low PTX concentrations just start the apoptotic signaling transduction pathway to cut out upstream of mitochondria permeabilization and do not cause ECs death [14].

DTX inhibits the migration of human umbilical vascular endothelial cells (HUVECs) and the proliferation of VSMCs and non-small-cell lung cancer cells, and induces mitochondria dysfunction, DNA damage, apoptosis and cell death of HUVECs [12, 16–19]. DTX promotes PKCβ phosphorylation and increases NADPH oxidase activity, which results in ROS formation. The modulation of PKCβ function plays an important role in DTX-mediated endothelial responses. DTX causes endothelial dysfunction through the PKCβ/NADPH oxidase pathway [18]. DTX increases apoptosis and G2/M cell cycle arrest in human renal clear cell carcinoma via suppressing mitogen-activated protein kinase signaling [20].

*In vitro*, DTX inhibits VEGF-induced HUVECs migration at concentrations lower than required to lead to cell-cycle arrest or apoptosis. DTX causes the separateness of Hsp90 from tubulin and leads to the ubiquitination and succedent proteasomal degradation of Hsp90 in HUVECs, which stops signaling from the VEGF receptors and focal adhesions and restrains integrin activation. DTX also decreases the VEGF-induced phosphorylation of Hsp90 client proteins, including focal adhesion kinase (FAK), Akt, and endothelial nitric oxide synthase (eNOS), and inhibits VEGF-induced eNOS activity [19]. DTX promotes microtubule assembly and stabilizes certain polymers against depolymerization, thereby inhibiting microtubule dynamics [11]. DTX efficiently decreases EC motility via interference with microtubule dynamics restraining the activation of Rac1/Cdc42 and disturbing the actin cytoskeleton [16]. For many cellular processes in particular cell migration, the skeleton network, including actin filaments, intermediate filaments and microtubules, is important.

DTX, as a candidate drug of DES, can efficiently inhibit proliferation of VSMCs [12]. But DTX-induced EC dysfunction is not fully understood. To better understand the probable effect of DTX toward HUVECs, using HUVECs (EA.HY926) as a cell model *in vitro*, we analyzed the effects of DTX on the proliferation, apoptosis, adhesion, migration and morphology of HUVECs. Further, we explored the signaling pathway that DTX inhibited HUVECs migration.

## Materials and methods

### Cell culture and reagents

The HUVEC line EA.Hy926 was obtained from American Type Culture Collection (ATCC, Manassas, VA). HUVECs were cultured with complete culture medium (RPMI 1640 medium supplemented with 10% fetal bovine serum (FBS), 1% penicilin–streptomycin, 1% glutamine) and maintained at 37°C with 5% CO_2_. Trypsin-EDTA was used to passage cells. All above agents were obtained from Gibco (Grand Island, New York, USA).

DTX was purchased from Xieli Pharmaceutical (Chengdu, China). CCK-8 kit and Annexin V-FITC Apoptosis Detection kit were purchased from Dojindo Laboratories (Shanghai, China) and KeyGEN Biotechnology (Nanjing, China), respectively. Anti-proliferative cell nuclear antigen (anti-PCNA) and anti-p53 were obtained from Proteintech Group (USA). Anti-β-actin, anti-GAPDH, anti-PECAM-1 and anti-integrin β1 were purchased from Santa Cruz Biotechnology (California, USA). Anti-VE-cadherin, anti-FAK, anti-pFAK, anti-Rho, anti-ROCK, anti-MLC2 and anti-pMLC were purchased from Abcam (Cambridge, UK). Anti-p190 RhoGEF was purchased from Bioss (Beijing, China). Y-27632 was purchased from Selleck chemicals (USA). HRP-conjugated and FITC/PE-conjugated anti-mouse/rabbit/goat secondary antibodies were obtained from Biosynthesis Biotechnology (Beijing, China).

### Investigation of cell proliferation and apoptosis

DTX powder was dissolved in DMSO and added to RPMI 1640 medium as the stock solution (5 μg/ml). The maximum final concentration of DMSO was <0.05%. For the investigation of cell proliferation, HUVECs were seeded into 96-well culture plates at 1 × 10^4^ cells/well and then cultured in RPMI 1640 medium containing 10% FBS at 37°C until 40–50% confluence. The medium was then replaced with serum-free medium. After cells were serum starved for 4 h, DTX was configured with a range of 0.05, 0.5, 5 and 50 nM. HUVECs were treated with the indicated DTX dosages. HUVECs without DTX served as the control group. Cell viability was assessed with the CCK-8 kit assay after incubation for 24, 48 and 72 h, respectively. In brief, CCK-8 (5 µl/well) was added to the culture medium and incubated at 37°C for 2 h. Absorption values were measured at 450 nm with a microplate reader. Inhibition rate (%) = (the OD value of treated group−OD value of blank group)/(the OD value of control group− OD value of blank group) × 100%. For the investigation of cell apoptosis, HUVECs were seeded into 6-well culture plates at 3 × 10^5^ cells/well and then cultured in RPMI 1640 medium containing 10% FBS at 37°C until 70–80% confluence. After the medium was replaced with serum-free medium, HUVECs were dosed with the indicated DTX dosages for 24 h. The apoptotic cells were assessed by flow cytometry using Annexin V-FITC Apoptosis Detection kit.

### Investigation of cell adhesion

HUVECs were seeded into 48-well culture plates at 1 × 10^4^ cells/well and then complete culture media with the indicated DTX dosages (0.05, 0.5, 5 and 50 nM) were added. HUVECs without DTX served as the control group. The plates were incubated at 37°C with 5% CO_2_. The samples were observed under the inverted microscope (OLYMPUS X-81, Japan) after culture for 0.5, 1, 2 and 4 h, respectively. Under the microscope, cell adhesion is the process converting a cell from a small bright spot to a solid black spot. Therefore, the solid black spots are adherent cells. The total number of cells and the numbers of adherent cells from 10 fields were counted. The cell adhesion rate (%) = the numbers of adherent cells/the total number of cells × 100%.

### Assay for directed migration of monolayer wounding

The spreading and migration capabilities of HUVECs were assessed using a scratch wound assay by measuring the expansion of a cell population on surfaces. The assay was performed as previously described with minor modification [21]. Cells were grown in a 6-well culture plate until nearly confluent cell monolayer. After cells were serum starved in media containing only 1% serum for 4** **h, a linear wound was established with a sterile 10** **μl plastic pipette tip in the confluence cell monolayer. The plates were rinsed twice with phosphate buffer saline (PBS). After adding the serum-free culture media containing a range of DTX concentration, the plates were incubated at 37°C with 5% CO_2_. To estimate the relative migration cells, the images from each scratched areas under each DTX concentration were taken for 0, 6, 12, 24 and 36** **h, respectively. Images were imported into Image J and converted to stacks for determining the degree of wound repair and migration distance.

### Assay for migration of transwell chamber

HUVECs migration was determined by a modified transwell chamber (Millipore) assay. The assay was performed as previously described with minor modification [22]. Prior to the experiment, the upper wells of each chamber were pre-coated with RPMI 1640 culture medium and incubated at 37°C for 30 min to wet PET membrane. Two hundred microliters of cell suspension (2 × 10^5^ cells/ml in complete culture medium) was added to the upper well and 600 μl of complete culture medium was added to the bottom well. The samples were maintained at 37°C with 5% CO_2_ for 4 h to allow cells adhesion on the PET membrane. Then the culture media in the upper wells were replaced with the serum-free culture media with the specified concentration of DTX, and then the samples were incubated for an additional 24 h. Cells were removed from the upper membrane surface by swabbing, and migrated cells (lower surface) were fixed with 95% alcohol. After air dry in ventilated place, the samples were stained with 0.1% crystal violet at room temperature for 30 min. Finally, the samples were observed under the microscope after being rinsed twice with PBS. The numbers of cells from ten fields were counted.

### Assay for migration, spreading area and shaft length of individual cells

The migration of individual cells was determined by living cells workstation [22]. Cells were seeded in culture dish for 12 h. After cells adhered, the original medium was removed and new media with the indicated DTX dosages, 1.5% FBS, penicillin-streptomycin and 1% glutamine were added. The culture dishes were placed in living cell workstation for 8 h. For each sample, 10 fields with one or two cells were selected, and time-lapse images of cells at each visual field location were taken at intervals of 5 min for 8 h. Finally, there were 96 pictures in each field. Images were imported into Image J and converted to stacks for determining the migration distance, spreading area and shaft length of individual cells.

### Immunofluorescence

Three hundred microliters of cell suspension (2 × 10^4^ cells/ml) was seeded into a 24-well culture plate until 50% confluence, and then complete culture media with the indicated DTX dosages were added. The plates were incubated at 37°C with 5% CO_2_ for 24 h. The subsequent immunofluorescence operation process was consistent with past [23]. Cells were fixed with 4% paraformaldehyde and permeabilized with 0.1% Triton-X 100. Cells were incubated with 1:100 dilution rabbit monoclonal anti-PCNA overnight at 4°C in wet box before block nonspecific staining with 1% BSA. After being rinsed three times with PBS and added with FITC-conjugated secondary antibodies (1:500), cells were kept in a dark place for 1 h at room temperature. Nuclear staining was performed with DAPI. The fluorescence of protein was observed by the fluorescence microscope.

### Western blot analysis

The immunoblotting of HUVECs was performed as previously described [12]. In brief, 300 μl of cell suspension (2 × 10^4^ cells/ml in RPMI 1640) was seeded into a 24-well culture plate and cultured at 37°C until 70–80% confluence. After starved in serum-free medium for 4 h, medium was replaced by the new complete culture medium with the indicated DTX dosages. The plates were incubated at 37°C with 5% CO_2_ for another 24 h. Radio-immunoprecipitation assay (Biyuntian) was used to extract total lysate. Proteins were transferred to PVDF membranes (Amersham Pharmacia Biotech) after the proteins were separated by electrophoresis on a 12% SDS-polyacrylamide gel electrophoresis. The membranes were blocked in TBST blocking buffer on shaker (50 rpm/min) for 2 h at room temperature. Then, they were incubated overnight at 4°C with the different dilution ratio of primary antibodies: GAPDH (1:1000), β-actin (1:1000), PCNA (1:1000), P53 (1:1000), VE-cadherin (1:50), PECAM-1 (1:500), integrin β1 (1:500), FAK (1: 1000), pFAK (1:1000), p190 RhoGEF (1:100), Rho (1:1000), ROCK (1:1000), MLC2 (1:1000), pMLC (1:2000), sequentially followed by the hybridization with HRP-conjugated secondary antibodies (1:5000–1:10 000) on shaker (50 rpm/min) for 1 h at room temperature. The expression of proteins was detected using ECL plus kit (Millipore), and the intensities were quantified by using Scion Image Software analysis.

### Statistical analysis

The experimental results are expressed as the means ± SD of three independent analyses. Statistical analyses were performed using GraphPad Software 5.0 (INSTAT, Graph Pad Software, Sorrento Valley, CA, USA). *P* < 0.05 is considered to be significant difference and *P* < 0.01 indicates extremely significant difference, “*” and “**” shows difference between the experimental group and control group, “#” and “##” indicates difference between treated groups.

## Results

### DTX inhibits cell proliferation and induces cell apoptosis in HUVECs

**Figure 1:**
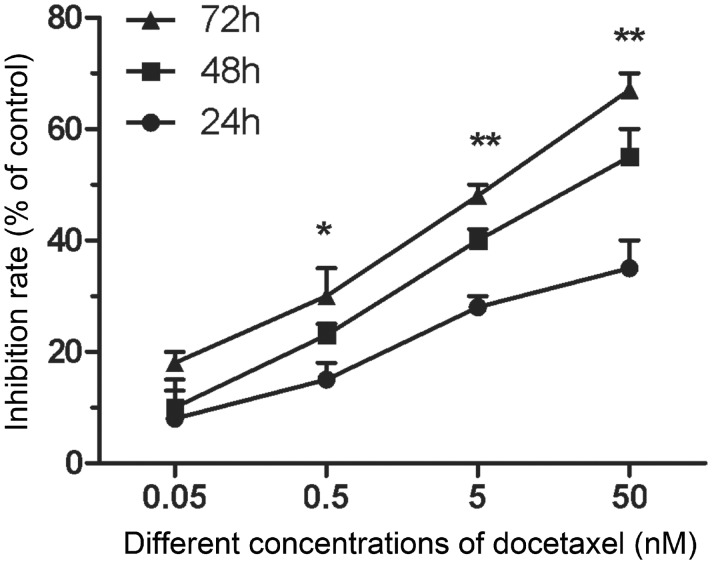
DTX inhibits HUVECs proliferation. The data represent the means of three independent experiments ± standard deviations (SD). *P < 0.05; **P < 0.01

To evaluate whether DTX inhibits HUVECs proliferation, CCK-8 assay was use to assess cell viability. The cell inhibition rates were quantitatively analyzed with GraphPad Prism 5.0 software. Compared with control group, the cell inhibition rates increased gradually with the increase of DTX concentration. After 24 h, the inhibition rates of DTX at 0.05, 0.5, 5 and 50 nM were 9.33, 14.33, 27.67 and 35%, respectively. In addition, the cell proliferation inhibition also enhanced with the extension of time. After 48 and 72 h, the inhibition rates of DTX at 0.05 nM were 10.67 and 17.67%, respectively; while the inhibition rates of DTX at 50 nM were 53.33 and 62.33%, respectively (Fig. 1). The results suggested that DTX inhibited cell proliferation in a concentration- and time-dependent manner.

**Figure 2:**
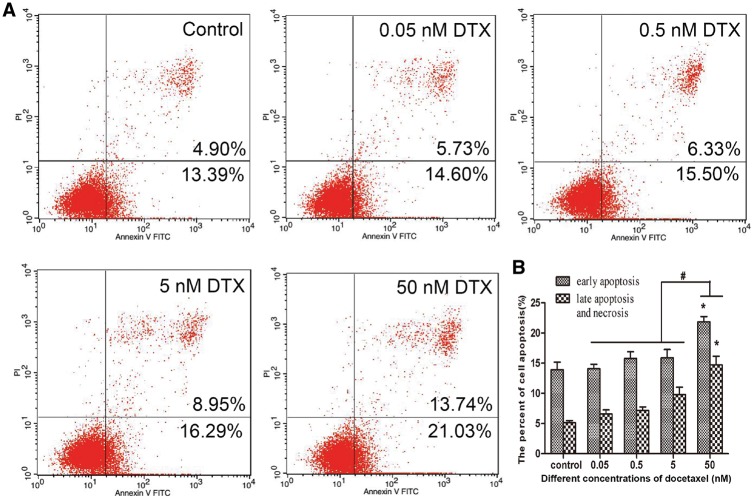
DTX leads to HUVECs apoptosis. **(A)** Flow cytometry test results. **(B)** The quantitative analysis based on (A). HUVECs were treated with DTX for 24 h. The data represent the means of three independent experiments ± SD; *P < 0.05 versus control. ^#^*P* < 0.05 versus 50 nM group

The flow cytometry was used to test whether DTX induces HUVECs apoptosis. Annexin-V FITC and PI were used to mark early and late apoptotic cells, respectively. After culture for 24 h, the early and late apoptotic rates in the control group were 13.39 and 4.90%, respectively. While those in the presence of DTX at 0.05, 0.5, 5 and 50 nM were 14.60–21.03 and 5.73–13.74%, respectively. The numbers of early and late apoptotic cells in the presence of DTX at 0.5 and 5 nM slightly increased when compared with that of the control group, but without significant difference. However, the early and late apoptotic rates in 50 nM DTX group significantly increased when compared with other groups (Fig. 2). In addition, there were a small number of visible floating cells under a microscope after 50 nM DTX treatment for 48 h (data not shown). The findings suggested that the high dosage of DTX (50 nM) showed a strong toxicity and promoted the cell apoptosis and necrosis after 24-h treatment. Therefore, the cytotoxicity of DTX is associated with dose and treatment time.

**Figure 3:**
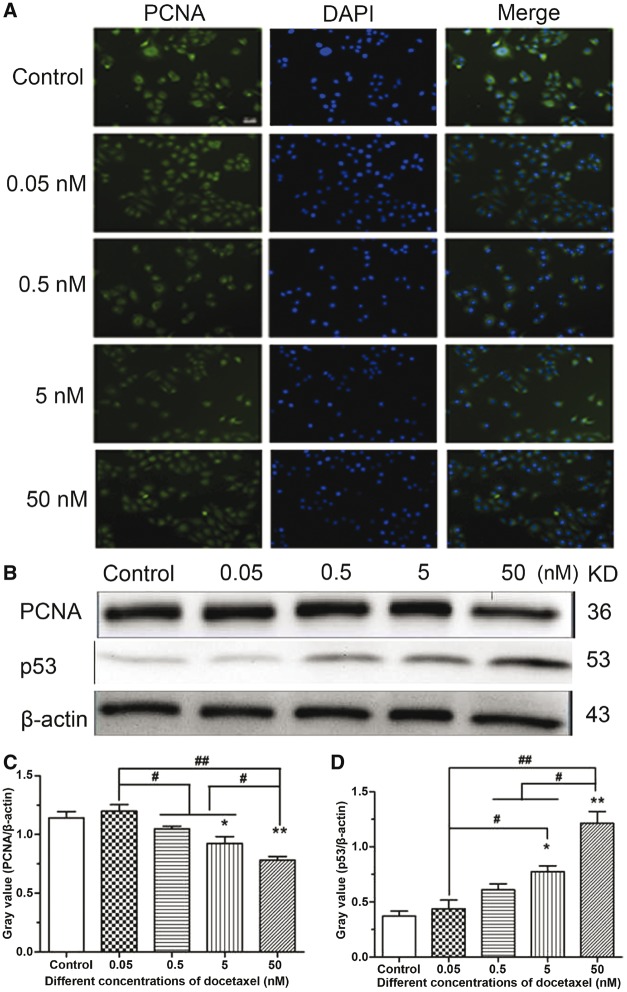
The expression analysis of PCNA and p53 in HUVECs. **(A)** PCNA immunofluorescence in HUVECs, standard magnification is 40×, and bar is 100 µm. **(B)** Western blot analysis. **(C**, **D)** The quantitative analysis of the gray value based on (B). HUVECs were treated with DTX for 24 h. The data represent the means of three independent experiments ± SD. *P < 0.05; **P < 0.01 versus control. ^#^*P* < 0.05; ^##^*P* < 0.01 versus between experimental groups

To confirm the antiproliferative activity of DTX toward HUVECs, the expression of proliferating cell nuclear antigen (PCNA) was measured by PCNA immunofluorescence and western blotting analysis. After HUVECs were incubated with various concentrations of DTX for 24 h, the fluorescence intensity weakened with the increase of DTX concentration, which indicated that DTX inhibited PCNA expression in a dose-dependent manner (Fig. 3A). The results of western blot analysis also showed that the expression of PCNA weakened with the increase of DTX concentration (Fig. 3B and C). The findings suggested that DTX had antiproliferative activity toward HUVECs. To confirm the pro-apoptosis activity of DTX toward HUVECs, The expression of p53 was analyzed by western blot analysis. The results are shown in Fig. 3B and D, after 24-h incubation, the expression of p53 strengthened gradually with the increase of DTX concentration. The findings suggested that DTX induced cell apoptosis by increasing the levels of p53.

### DTX delays HUVEC adhesion

**Figure 4:**
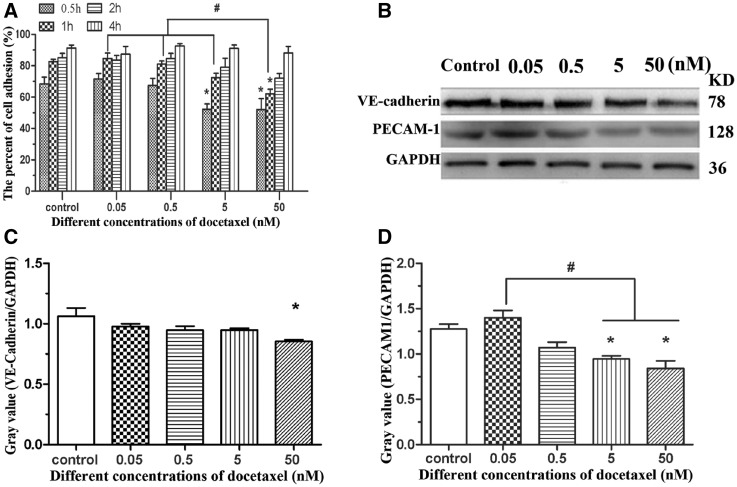
DTX delays HUVECs adhesion. **(A)** HUVECs adhesion. **(B)** Western blot. **(C**, **D)** The quantitative analysis of the gray value based on (B). HUVECs were treated with DTX for 24 h. The data represent the means of three independent experiments ± SD. *P < 0.05 versus control. ^#^*P* < 0.05 versus between experimental groups

To evaluate whether DTX inhibits HUVEC adhesion, the cell adhesion rates were calculated after culture for 0.5, 1, 2, and 4 h, respectively. After 0.5-h incubation in culture media without DTX and with 0.05, 0.5, 5 and 50 nM DTX, the cell adhesion rates were 68.52, 72.53, 67.34, 52.43 and 51.97%, respectively. Compared with control group, the adhesion rates in 0.05 and 0.5 nM DTX groups had no significant difference, but significantly decreased in 5 and 50 nM DTX groups. After 1-h incubation, the cell adhesion rates in 0.05, 0.5 and 5 nM DTX groups had no significant difference compared with control group, but the cell adhesion rate in 50 nM DTX was lower than that of other groups. After 2-h incubation, the cell adhesion rate in 50 nM DTX was slightly lower than that of other groups. After 4-h incubation, there was no significant difference among these five groups; their cell adhesion rates were 88.47–92.87% (Fig. 4A). The findings showed that DTX could delay HUVEC adhesion.

To confirm the anti-adhesive activity of DTX toward HUVECs, the expression of adhesion protein VE-cadherin and PECAM-1 from HUVECs was analyzed by western blot analysis after 24-h incubation. Compared with the control group, the expression of VE-cadherin and PECAM-1 was inhibited by DTX in a dose-dependent manner (Fig. 4B–D). The results of western blot analysis also showed that DTX had anti-adhesive activity toward HUVECs.

### DTX inhibits the migration of group of HUVECs

Because DTX at 50 nM had obvious toxicity to HUVECs, low doses of DTX at 0.05, 0.5 and 5 nM were used. The effects of DTX on HUVECs migration and invasion were investigated using scratch experiment and transwell chamber method, respectively.

**Figure 5:**
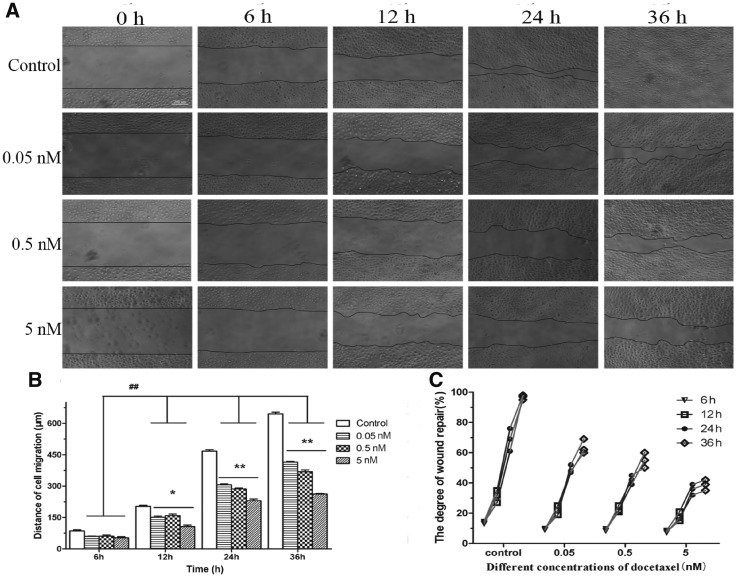
DTX inhibits HUVECs migration by scratch experiment. **(A)** The microscope images of cell migration, standard magnification is 40×, bar is 200 µm, and the black line is the front of cell migration. **(B)** The quantitative analysis of migration distance based on (A). **(C)** The quantitative analysis of wound healing degree based on (A). The data represent the means of three independent experiments ± SD. *P < 0.05; **P < 0.01 versus control. ^##^*P* < 0.01 versus between experimental groups

The scratch-wounded HUVECs monolayer was incubated with or without DTX (Fig. 5). In Fig. 5A, the black line was the front of cell migration at injury site. The images were imported into Image J and converted to stacks for determine degree of migration. After the presence of DTX at 0.05, 0.5 and 5 nM for 6–36 h, the distances of HUVECs migration were 60.00–415.00, 60.67–369.00 and 53–262.33 µm, respectively, while in the absence of drug, the distances of HUVECs migration were 86.67–644.67 µm (Fig. 5B). The degrees of wound repair in the presence of DTX at 0.05, 0.5 and 5 nM for 6–36 h were 9.30–63.67, 8.83–55.00 and 7.87–38.67%, respectively, while those of the control group were 13.77–96.67% (Fig. 5C). The findings indicated that the migration and wound repair of HUVECs were inhibited by DTX in a time- and concentration-dependent manner.

**Figure 6:**
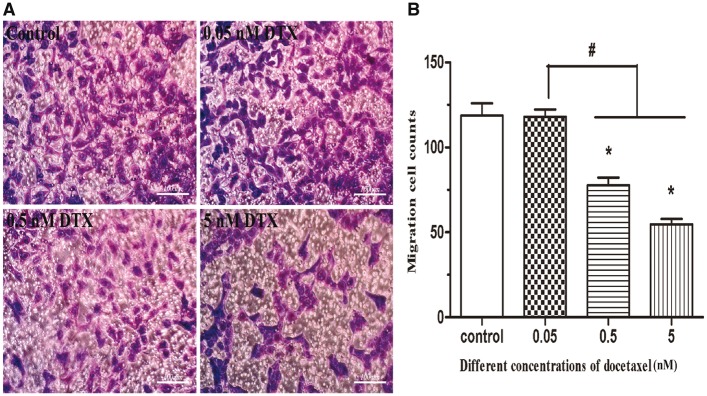
DTX inhibits HUVECs migration by transwell chamber method. **(A)** The microscope images of cell migration, standard magnification is 100×, and bar is 100 µm. **(B)** The quantitative analysis according to the average number of cell migration. HUVECs were treated with DTX for 24 h. The data represent the means of three independent experiments ± SD. *P < 0.05 versus control. ^#^*P* < 0.05 versus between experimental groups

The results of cell invasion are shown in Fig. 6. After the presence of DTX at 0.05, 0.5 and 5 nM for 24 h, the numbers of invasive cells were 118, 78 and 55, respectively, while the number of the control group was 119. The numbers of invasive cells in the presence of DTX at 0.5 and 5 nM were significantly lower than those in DTX treatment group at 0.05 nM and control group, indicating that the dosage of DTX ≥0.5 nM could significantly inhibit the invasion of HUVECs. This conclusion is similar to the scratch experiment.

### DTX suppresses the migration of individual HUVECs and decreased their spreading area and aspect ratio

**Figure 7:**
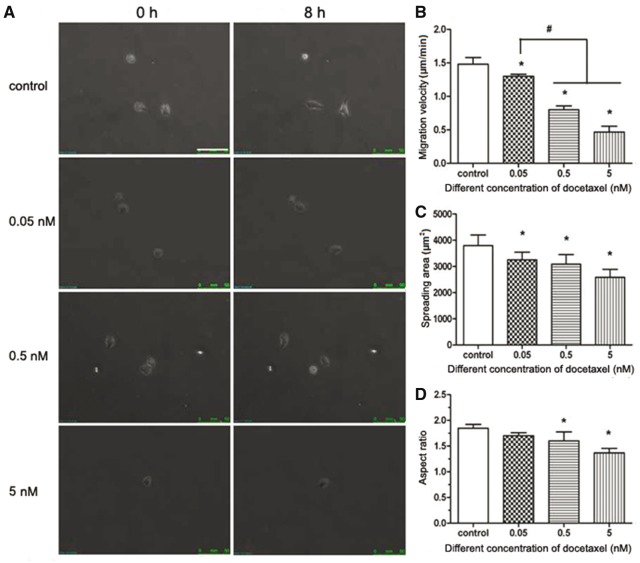
DTX inhibits the migration and spread area of individual HUVEC. **(A)** The fluorescence microscope images of cell movement, standard magnification is 100×, and bar is 100 µm. **(B)** Cell migration velocity. **(C)** Cell spreading area. **(D)** Cell aspect ratio. HUVECs were treated with DTX for 8 h. The data represent the means of three independent experiments ± SD. *, *P* < 0.05 versus control. ^#^*P* < 0.05 versus between experimental groups

The microscope photos were obtained after HUVECs were treated with DTX for 8 h (Fig. 7A). The images were imported to Image J software to determine the spreading area and length of long axis and short axis of the cell and the center coordinate (used to analyze cell migration path and direction) of individual cell of each images. Quantitative analysis results are shown in Fig. 7B–D. In the control group and in the presence of DTX at 0.05, 0.5 and 5 nM for 8 h, the migration rates were 1.48, 1.30, 0.80 and 0.47 µm/min, respectively (Fig. 7B), the average spreading areas of individual HUVEC were 3793.33, 3250.00, 3083.33 and 2583.33 µm^2^, respectively (Fig. 7C), and the aspect ratios were 1.85, 1.70, 1.69 and 1.36, respectively (Fig. 7D). The results showed that the migration rate, spreading area and aspect ratio of individual HUVECs in all DTX treatment groups were significantly lower than those in the control group, and the DTX inhibited the migration of individual HUVECs and decreased their spreading area and aspect ratio in a dose-dependent manner.

### DTX suppresses the expression of integrin β1, p190 RhoGEF, Rho and ROCK, and reduces the phosphorylation levels of FAK and MLC in HUVECs

**Figure 8:**
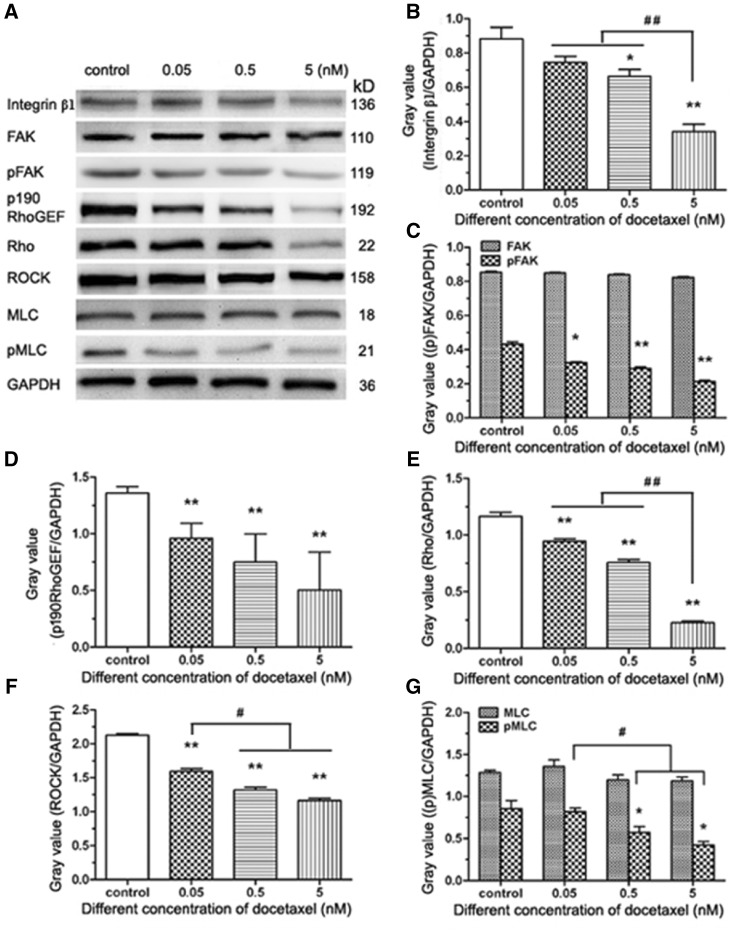
The western blot analysis of integrin-mediated FAK/ROCK signaling pathway in HUVECs. **(A)** Western blot analysis. **(B–G)** The quantitative analysis of the gray value based (A). HUVECs were treated with DTX for 24 h. The data represent the means of three independent experiments ± SD. *P < 0.05; **P < 0.01 versus control. ^#^*P* < 0.05; ^##^*P* < 0.01 versus between experimental groups

Integrin-mediated signaling pathway requires recruiting many intracellular proteins to the focal adhesion, including FAK, a vital regulator of motility and adhesion in cells. To verify whether DTX inhibits HUVECs migration by integrin-mediated FAK/ROCK signaling pathway, integrin β1, FAK, pFAK, p190 RhoGEF, Rho, ROCK, MLC and pMLC were analyzed by western blot analysis (Fig. 8A). The data showed that the expression of integrin β1, p190 RhoGEF, Rho and ROCK was inhibited significantly by DTX in a dose-dependent manner (Fig. 8B, D–F). The expression of FAK and MLC was not affected by DTX, but the contents of pFAK and pMLC decreased with the increase of DTX concentration. The results showed that the phosphorylation levels of FAK and MLC were suppressed by DTX in a concentration-dependent manner (Fig. 8C and G).

### DTX and Y-27632 synergistically inhibit HUVECs migration and reduce the contents of ROCK and pMLC in HUVECs

**Figure 9:**
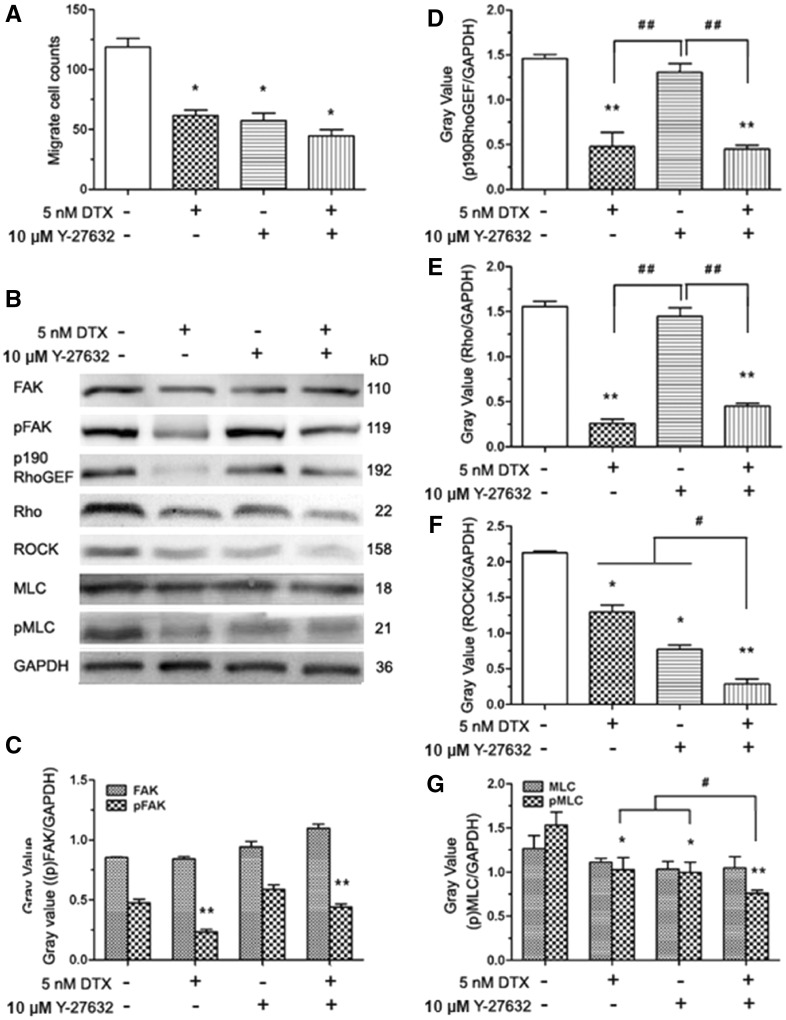
DTX and Y-27632 inhibits HUVECs migration and the western blot analysis in HUVECs. **(A)** The number of cell migration. **(B)** Western blot analysis. **(C–G)** The quantitative analysis of the gray value based (C). HUVECs were pro-treated 2 h with Y-27632 prior to DTX for 24 h. “+” means added a substance, “−” means no join; the data represent the means of three independent experiments ± SD. *P < 0.05; **P < 0.01 versus control. ^#^*P* < 0.05; ^##^*P* < 0.01 versus between experimental groups

The Y-27632 that is the specific ROCK1/2 inhibitor was used to block the phosphorylation and activity of the downstream effectors in the Rho signaling pathway. HUVECs were pre-treated with Y-27632 for 2 h, the cells became smaller and boundaries clear between cytoplasm and nuclear under the microscope (data not shown), which showed the suppressive effects of Y-27632 on cell cytoskeleton. HUVECs migration analysis and western blot analysis are showed in Fig. 9. The average numbers of migration cells in the absence of drug and in the presence of DTX, Y-27632 and DTX plus Y-27632 were 119, 62, 57 and 39, respectively. The results showed that the numbers of migration cells of the experimental groups with DTX and/or Y-27632 significantly reduced compared with the control group, while the number of migration cells in the DTX plus Y-27632 treatment group was lower than those of the DTX or Y-27632 treatment group (Fig. 9A). Western blot analysis showed that the contents of FAK and MLC did not have statistically significant difference between the experimental groups with DTX and/or Y-27632 and the control group (Fig. 9B, C and G), the contents of pFAK, p190 RhoGEF, Rho, ROCK and pMLC in DTX treatment group were significantly lower than those of the control group (Fig. 9B–9G). Although the contents of pFAK, p190 RhoGEF, Rho and MLC did not have statistically significant difference between Y-27632 treatment group and the control group (Fig. 9B–E and G), the contents of ROCK and pMLC in Y-27632 treatment groups were significantly lower than those of the control group (Fig. 9B, F and G). In addition, the contents of ROCK and pMLC in the DTX plus Y-27632 treatment group were lower than those of the DTX or Y-27632 treatment group (Fig. 9B, F and G). The findings indicated that DTX and Y-27632 synergistically inhibited HUVECs migration by reducing the contents of ROCK and pMLC in HUVECs (Fig. 9B, F and G).

## Discussion

The angiogenesis is inherent to normal growth and development, as well as to wound healing. ECs release factors that control vascular relaxation and contraction, thrombogenesis, fibrinolysis and platelet activation and inhibition [24]. DTX has broadly been used as an antiangiogenesis drug. However, DTX causes some side effects, such as vessel damage and phlebitis, which may reduce its clinical therapeutic efficacy. DTX induces the apoptosis and death of HUVECs and inhibits HUVECs migration [16, 18, 19]. In this study, DTX not only induced the apoptosis and necrosis of HUVECs and inhibited migration of HUVECs, but also inhibited HUVEC proliferation and delayed adhesion and reduced the spreading area of HUVECs. These side effects are not conducive to the endothelialization of injured endothelium. DTX’s cytostatic effect at low concentrations and the cytotoxic effect at high concentrations are consistent with PTX [14, 15].

The antiproliferative effect of DTX on VSMCs and mechanism had been investigated. The antiproliferative activity of DTX is related to cell cycle arrest in the G0/G1 phase, which may be due to the inhibition of CDKs/cyclins complex expression and the decrease of retinoblastoma protein (pRb) phosphorylation [12, 25]. The inhibition of pRb phosphorylation by DTX is correlated significantly with the suppression of DNA synthesis and cell cycle progression [26]. PCNA is a gene product induced by phospho–Rb in VSMCs, which is also inhibited by DTX [12]. The expression of PCNA is consistent with the synthesis of DNA during cell proliferation, which can be used to evaluate cell proliferation [12, 27]. The present results indicated that the expression of PCNA decreased with increase of DTX concentration in HUVECs, suggesting that DTX implements its antiproliferative effect. The findings are consistent with the previously experimental results obtained from VSMCs [12].p53 transcription factor, a tumor suppressor protein, plays a key role in coordinating cell cycle arrest and apoptosis in response to various cellular stresses. The expression of p53 represents cells apoptosis [28, 29]. Therefore, we focused on confirming whether the DTX affected the p53 expression in DTX-induces HUVECs apoptosis. The present experimental data showed that the expression of p53 increased with the increase of DTX dosages, which further demonstrates that DTX promotes apoptosis in a concentration-dependent manner.

In ECs, the adherens junctions largely consist of VE-cadherin, which is an endothelium-specific member of the cadherin family. VE-cadherin is a single-span transmembrane protein expressed by ECs and its extracellular domain forms homomeric dimers with VE-cadherin molecules of adjacent cells [30]. Several adhesion proteins, including PECAM-1, MUC18, ICAM2, CD34, endoglin and others, cluster at cell–cell contacts [31]. Expression of VE-cadherin and PECAM-1 enhanced the adhesion of acute lymphoblastic leukemia to brain-derived microvasculature ECs [32]. The sustained activation of MAPK/ERKs led to down-regulation of PECAM-1 expression, disruption of cadherin-mediated cell–cell adhesion [33]. The expression of VE-cadherin and PECAM-1 affects EC adhesion and the function of ECs via downstream signaling pathways. The microtubules destroy drugs may influence vascular morphogenesis by disturbing the interaction of adjacent ECs, possibly as a result of effects on VE-cadherin, β-catenin and/or actin [34]. The results of this investigation showed that DTX could inhibit the expression of VE-cadherin and PECAM-1 and delay HUVEC adhesion, indicating that DTX delayed HUVEC adhesion via down-regulation of VE-cadherin and PECAM-1 expression.

Adhesion, migration and invasion are important for HUVECs metastasis. The migration and invasion of HUVECs play an important role in the wound healing process of the intimal injury. The cytoskeleton confers the extent of integrin junction, allowing an adhesive contact to obtain enough strength to withstand contractile forces relating to cellular movement and function [35]. The crucial part of the adhesive function of integrins is the ability that connects to the actin cytoskeleton [35]. The activities of integrin β1 and FAK are important for the focal adhesion complex formation, which controls cell migration and adhesion in human glioblastoma [36]. The focal adhesion complex is directly associated with ROCK pathways [37]. FAK can boost cytoskeletal tension by the phosphorylation and activation of p190 RhoGEF. Succedent Rho activation indirectly modulates MLC phosphorylation via the ROCK phosphorylation of MLC phosphatase, which causes the increase of MLC kinase activity by down-regulating MLC phosphatase activity [38]. FAK–Rho–ROCK–(p)MLC–myosin signaling transduction pathway plays an important role in cell shape change, adhesion and migration [38, 39]. Therefore, we speculated that the DTX inhibits HUVECs migration, delays HUVECs adhesion and induces HUVECs shape change via the inhibition of integrin β1-mediated (p)FAK–Rho–ROCK–(p)MLC–myosin signaling transduction pathway. To prove this hypothesis, the related proteins were detected by western blot analysis. The results showed that DTX suppressed the expression of integrin β1, p190 RhoGEF, Rho and ROCK, and reduced the phosphorylation levels of FAK and MLC in HUVECs in a dose-dependent manner.

**Figure 10:**
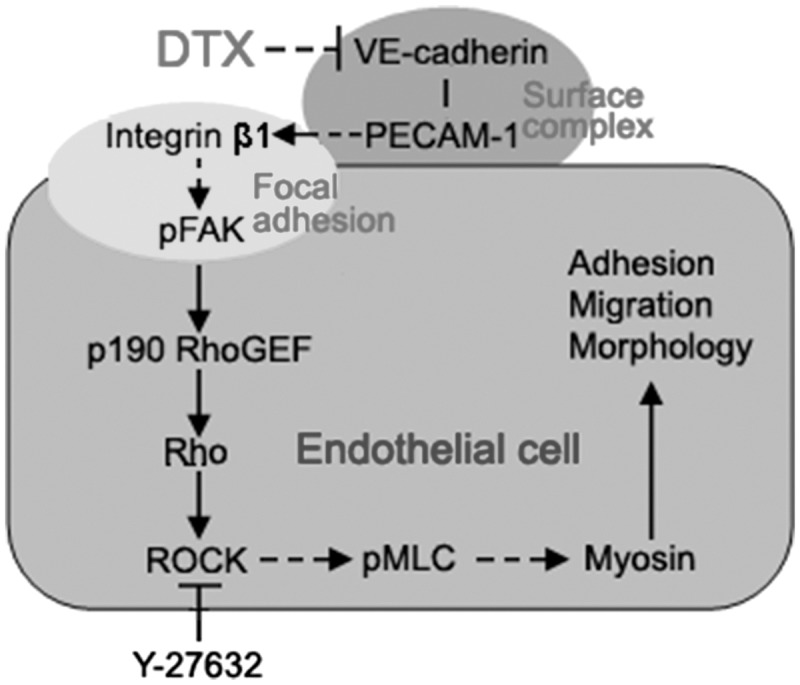
The schematic illustration of the DTX-induced inhibition of HUVECs migration through the VE-cadherin mediated integrin β1/FAK/ROCK signaling pathway. The arrow indicates activation or induction, and the (⊣) indicates inhibition or blockade

However, integrins can either stimulate or suppress actin-based structures in different contexts, indicating the various pathways leading from integrins to the cytoskeleton [35]. In glioma cells, caffeine inhibits migration by the ROCK–FAK pathway [40]. To confirm whether DTX inhibits HUVECs migration, delays HUVECs adhesion and induces HUVECs shape change via integrin β1-mediated ROCK–FAK pathway or integrin β1-mediated (p)FAK–Rho–ROCK–(p)MLC–myosin signaling transduction pathway, ROCK inhibitors Y-27632 was also used to define the signaling pathway. The result showed that the number of migration cells of the experimental groups with Y-27632 significantly reduced compared with the control group, and the contents of ROCK and pMLC in Y-27632 groups were significantly lower than that of the control group; but the contents of FAK, pFAK, p190 RhoGEF, Rho and MLC were not affected by Y-27632. In addition, DTX and Y-27632 synergistically inhibited HUVECs migration and reduced the contents of ROCK and pMLC in HUVECs. The results obtained showed that DTX inhibited HUVECs migration, delayed HUVECs adhesion and induced HUVECs shape change through integrin β1-mediated (p)FAK–Rho–ROCK–(p)MLC–myosin signaling transduction pathway rather than through the ROCK–FAK pathway. In addition, the surface complex of VE-cadherin, PECAM-1 and VEGFR2 is the major pathway to activate integrins [41]. DTX also inhibited the expression of VE-cadherin and PECAM-1 in this research, Therefore, DTX inhibits HUVECs migration, delays HUVECs adhesion and induces HUVECs shape change through VE–cadherin–PECAM-1–integrin β1–(p)FAK–p190 RhoGEF–Rho–ROCK–(p)MLC–myosin signaling pathway that is VE-cadherin-mediated integrin β1/FAK/ROCK signaling pathway (Fig. 10).

## Conclusion

DTX had the cytostatic effect at low concentrations and the cytotoxic effect at high concentrations. DTX could inhibit HUVECs proliferation, induce HUVECs apoptosis, delay HUVECs adhesion and decrease the spreading area and aspect ratio of individual HUVEC. DTX suppresses HUVECs migration, delays HUVECs adhesion and induces HUVECs shape change by decreasing the expression of VE-cadherin, PECAM-1, integrin β1, p190 RhoGEF, Rho and ROCK, and the phosphorylation levels of FAK and MLC in HUVECs. DTX and Y-27632 also synergistically inhibited HUVECs migration by reducing ROCK and pMLC in HUVECs. Together, the present study successfully illustrates that DTX reduces the migration of HUVECs, delays HUVECs adhesion and induces HUVECs shape change through VE-cadherin-mediated integrin β1/FAK/ROCK signaling pathway.

As a candidate drug of DES, the release concentration of DTX around the implant may have cytostatic or cytotoxic effect on ECs, which will lead to delay of re-endothelialization. This study found the cytostatic and cytotoxic threshold concentration of DTX for ECs, which will be beneficial to control the local concentration of DTX from implant release below the cytotoxicity threshold of ECs. Therefore, this study provides useful information for the future designing of DTX in DES. However, this research was performed *in vitro*. Thus, further confirmation *in vivo* is needed in the future.

## Funding

The study was supported by grants from the National Natural Science Foundation of China (11332003, 31370949 and 81400329), the National Key Technology R & D Program of China (2016YFC1102305 and 2012BAI18B02), the Chongqing Graduate Student Research Innovation Project (CYS14023), the Fundamental Research Funds for the Central Universities (106112016CDJXZ238802), as well as the support from the Chongqing Engineering Laboratory in Vascular Implants and the Public Experiment Center of State Bioindustrial Base (Chongqing), China. 

*Conflict of interest statement*. None declared.
